# Conservative Surgery

**Published:** 1882-10

**Authors:** P. W. Van Peyma


### Conservative Surgery.

By P. W. Van Peyma, M. D.

J. N., a boy of 16, while employed in the upper story of a planing mill, fell through the elevator opening, a distance of about sixteen feet. When seen, he was found to have received, in addition to other injuries of a more trivial character, a compound dislocation of the lower end of the right radius. By enlarging the opening through the skin this was readily reduced. In the course of the following two weeks serious inflammation and suppuration of the wrist and hand ensued, necessitating a number of incisions. Despite these, the suppuration continued, resulting in the rupture of a vein and consequent serious hemorrhage. At the end of three weeks from the time of injury, the condition of the patient, both local and general, was bad; the hand and wrist greatly swollen, the discharging pus far from laudable and the circulation of the parts decidedly impaired; the pulse being weak and rapid, the stomach irritable. A week later the condition being unimproved, an aneurism of the radial artery was observed developing, the onward course of which, in spite of compression, we were unable to check. A ligature applied cut through a few days later, and rupture occurring, the hemorrhage was controlled by the attendants by means of a tourniquet, which, as a precautionary measure, had been applied a number of days previous. Counsel was now called, and the search for the radial artery being futile, the hemorrhage was temporarily controlled by means of four strong ligatures, including most of the tissue anterior to the bone. Amputation to be performed the following morning was advised as affording the only hope for recovery. The following morning Dr. C. C. F. Gay was called in consultation and resection of the lower end of radius decided upon. This was readily accomplished, the only apprehension being the general condition of the patient, which was extremely critical. For the next few days, the question of life and death was very evenly balanced, and the convalescence which ultimately occurred was delayed for many weeks. At the time of this communication, however, the patient has for a considerable period been at work, earning his former wages, with a hand which, although considerably weakened, is in all other respects of great usefulness.

Two points of practical interest occur in reviewing this case. In the light of future events there can be no doubt that immediate resection would have been the better treatment. I was withheld from this by having recently seen a case of compound dislocation of the ankle joint result in a very useful joint after simple reduction.

Also the case calls attention to the advantages of conservative surgery. Had the advice of the counsel first called been followed, the boy, if alive, would still be seriously crippled in the long years to come. Why amputation should have been preferred, appears unaccountable, as the shock of the operation would have been at least as severe.

